# Supplementary material for: Opioid-Modulated Receptor Localization and Erk1/2 Phosphorylation in Cells Coexpressing μ-Opioid and Nociceptin Receptors
Source: Int J Mol Sci. 2023 Jan 5;24(2):1048. doi: 10.3390/ijms24021048 (PMC9865058; doi:10.3390/ijms24021048)
Supplement: Supplementary file 1 [file ijms-24-01048-s001.zip › ijms-2052014-supplementary.pdf]

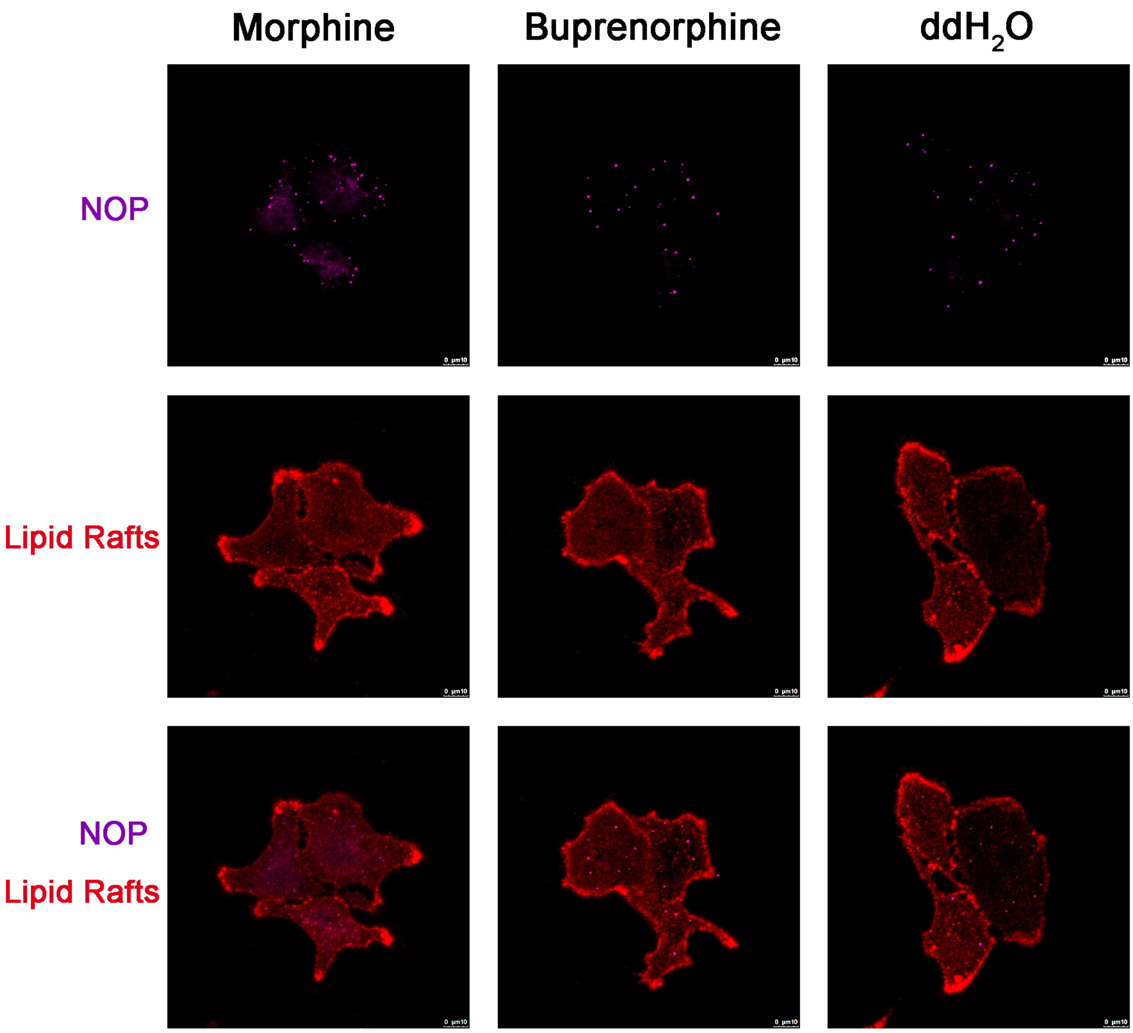

Figure S1. The individual channel images for NOP in Figure 3A, middle panels. (*Red*) lipid rafts, (*magenta*) myc-tagged NOP.
